# The impact of a health systems strengthening initiative on child morbidity: The case of the Ghana Essential Health Interventions Program in rural northern Ghana

**DOI:** 10.1371/journal.pone.0269199

**Published:** 2022-06-03

**Authors:** Patrick Opoku Asuming, Ayaga Agula Bawah, Edmund W. Kanmiki, James F. Phillips

**Affiliations:** 1 Department of Finance, University of Ghana Business School, Accra, Ghana; 2 Regional Institute for Population Studies, University of Ghana, Accra Ghana; 3 Institute for Social Science Research, University of Queensland, Indooroopilly, QLD, Australia; 4 Heilbrunn Department of Population and Family Health, Mailman School of Public Health, Columbia University, New York, New York, United States of America; Clinton Health Access Initiative, SOUTH AFRICA

## Abstract

**Background:**

Improving child and maternal health remains a core objective of global health priorities, extending from the millennium development goal (MDG) era to the current focus on the Sustainable Development Goals (SGDs). This paper analyses the childhood morbidity effects of the Ghana Essential Heath Interventions Program (GEHIP), a community-based health systems strengthening in rural northern Ghana. GEHIP was a five-year embedded implementation science plausibility trial that implemented a set of health systems strengthening strategies and tested the proposition that their combined effect at the district, subdistrict and community levels could foster effective community engagement and thereby improve maternal and child health outcomes.

**Methods:**

A two stage random sample survey of reproductive-aged women residing in treatment and comparison districts at the GEHIP baseline and end line was used for Heckman Difference-in-differences (DiD) regression models for estimating the incremental effect of GEHIP exposure on three child morbidity conditions (diarrhea, fever and cough), as recalled by maternal respondents in the course of survey interviews.

**Results:**

After controlling for child age and gender, maternal age, education, marital status, health insurance status, religion, ethnicity, occupation and household wealth index, regression results show that GEHIP had a statistically significant 45% reduction in fever (OR = 0.55, CI = 0.31–0.98) and 47% reduction in cough (OR = 0.53, CI = 0.30–0.94), over and above temporal reductions that prevailed in study districts. Although not significant, GEHIP also had 38% reduction in the incidence of diarrhea.

**Conclusion:**

Previous research has shown that GEHIP had a pronounced positive effect with a reduction in mortality. Our results show that household location in GEHIP districts also led to a significant reduction in morbidity due to cough and fever among under-five children. This association is a likely outcome of GEHIP’s impact on the accessibility of primary health care services. Results lend further support to the growing body of evidence that strengthening health systems in rural Africa through the provision of community-based strategies enhances prospects for achieving the United Nations child health SDGs.

## 1. Introduction

Despite an abundance of evidence on the causes of poor child health and wellbeing, most low and middle income countries failed to achieve the goals for childhood mortality reduction that the Millennium Development Goals embraced [1]. The current Sustainable Development Goals (SDGs) have continued to prioritize improvement in child and maternal health with the global goal of reducing under-five mortality to less than 25 per 1000 live births by the year 2030 [2]. The burden of childhood illness is higher in low and middle income countries (LMICs), underscoring the importance of implementing proven high-impact interventions in poor countries if the SDGs are to be achieved.

### 1.1. Health system development in Ghana

In recent years, Ghana has been at the forefront of community-based primary health care systems development in Africa. Policies that can be traced to the Alma Ata accord [3], refined by formative research in Navrongo over the 1994–1996 period [4, 5] and tested by a plausibility trial from 1996 to 2003 [6–10], showed that community-based primary health care can save childhood lives and reduce fertility. In response to this evidence, the Ghana Health Service (GHS) launched the Community-based Health Planning and Services (CHPS) Initiative in 1999 to scale-up lessons learned [11, 12]. While CHPS has been successful where it has been implemented, a variety of service delivery, manpower, communication, logistics, resource management, and leadership bottlenecks have constrained the pace of CHPS scale up [13–16].

In 2009, the Ministry of Health (MOH) launched a qualitative appraisal of the CHPS program which aimed to clarify operational factors that constrained CHPS scale-up. The review provided a basis for comparing leadership responses to questions about CHPS implementation in regions and districts where CHPS was progressing well with corresponding responses in regions and districts where the pace of implementation was unacceptably slow [17]. Results of this review provided an organizational diagnosis of systems development needs and an agenda for reform that informed the operational design of a project, known as the Ghana Essential Health Interventions Program (GEHIP) [18]. Launched in the Upper East Region (UER) in early 2010, and ending in December, 2015, GEHIP was embedded in the GHS regional program [19].

## 2. The GEHIP design

**Fig 1:**
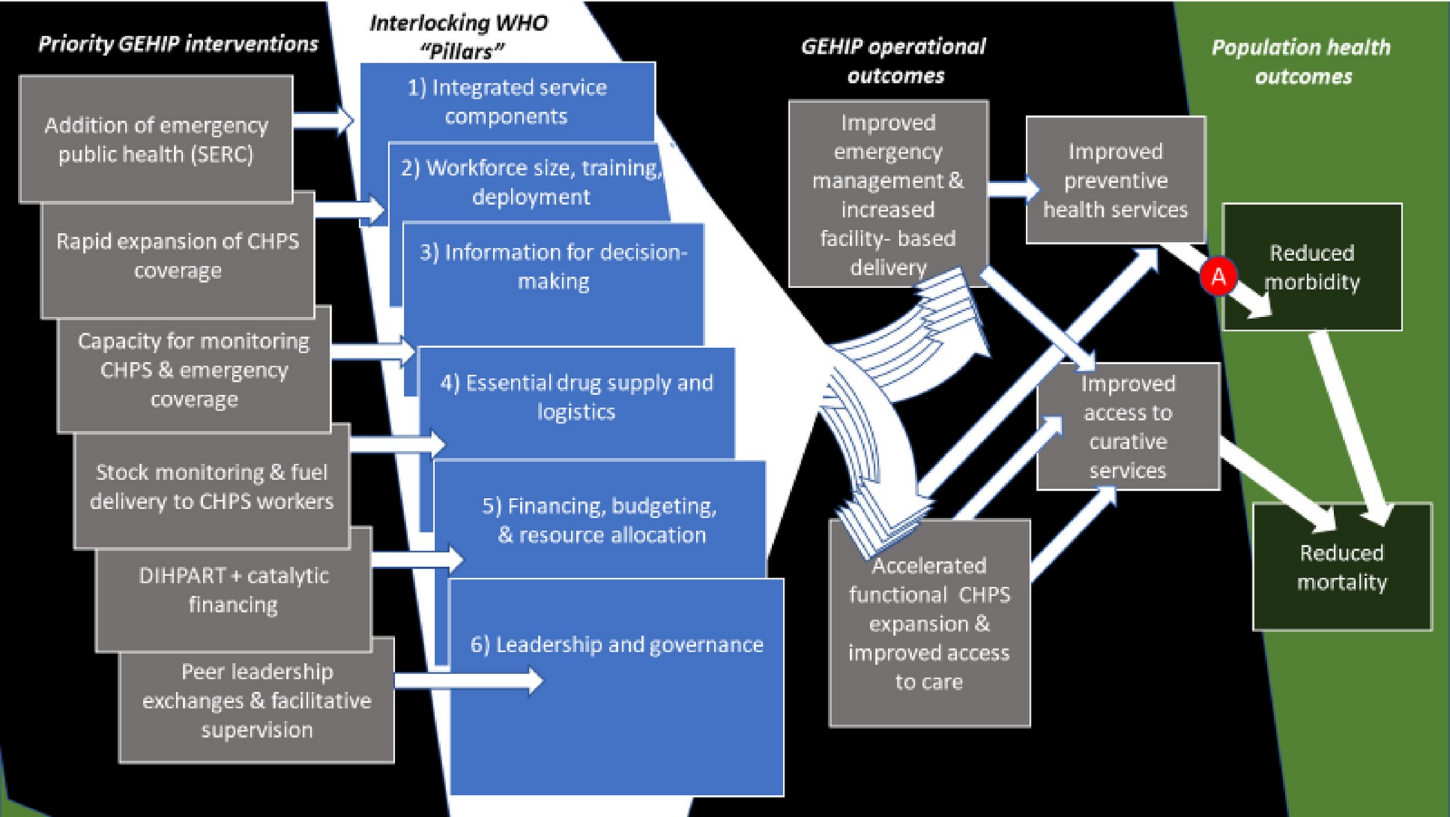
Pathway for the influence of health system strengthening on morbidity and mortality.

In 2007, the World Health Organization disseminated a summary report positing six sets of critical elements of effective health systems functioning [20]. GEHIP was designed to test the proposition that these building blocks, when strengthened at the district level, lead to improvements in health system functioning and eventually improve health and survival. Based on a preliminary appraisal of system problems and needs, six sets of targeted interventions were undertaken by GEHIP to strengthen the health system (Fig 1). All corresponded to WHO “pillars” of health system strengthening: i) Actions expanded the range of primary health care functionality by conducting community-engagement for organizing emergency referral systems and improving access to care by engaging volunteers to develop interim facilities for launching CHPS services. ii) Improving the capacity of manpower to provide primary health care was pursued by training volunteers to support the WHO “integrated management of childhood illness” programme [21], iii) Information systems were developed to improve the capabilities of Ghana Health Service (GHS) to monitor CHPS service coverage and functionality while simplifying and improving information systems for frontline workers. iv) Actions were undertaken to improve monitoring and management operations for assuring appropriate logistics and supplies. v) GEHIP developed a tool for budgeting that linked financial planning with the burden of disease profile, while engaging in dialogue and diplomacy essential for securing district development revenue for CHPS start-up costs. vi) Co-direction of operations was focused on redirecting district leadership training to emphasize community engagement, governance, and district resource mobilization. This package of activities was focused on four treatment districts in Ghana’s Upper East Region (UER) of northern Ghana. Seven contiguous UER districts served as GEHIP comparison areas.

While implementation of system-based primary health care at the community level predates GEHIP, the GEHIP interventions were implemented as a response to a Ministry of Health (MoH) commissioned review report that documented critical bottlenecks impeding successful scale up and implementation of CHPS [17]. Bottlenecks that were identified included a lack of district leadership understanding of CHPS implementation milestones and procedures, a tendency of managers and supervisors to ignore the need for community-engagement for implementing and operating CHPS, and a gradual, but relentless, institutional drift of community health services from its community engaged origins to a static, clinical service program. As a consequence, community-based resources for supporting implementation were lost. In particular, volunteer sponsored construction of interim community facilities represented a strategy for starting services without delays that are associated with financing and progressing with permanent facility construction. In the absence of budget-lines and procedures for covering start-up costs, district managers faced unacceptable delays in launching CHPS. Yet, there had been extensive investment in worker recruitment and training. Manpower for CHPS is developing, without supporting investment in facilities, equipment, supervision, or leadership development. GEHIP was developed to respond to these bottlenecks to reorient CHPS implementation to achieve its original desired results.

As Fig 1 illustrates, interlocking GEHIP interventions spanned the six WHO systems strengthening pillars. Outcomes were related to the impact of developing emergency referral care [22] and the impact of improving access to community-based primary health care. These outcomes, in turn, have sets of preventive and curative health outcomes that affect morbidity and mortality. As Fig 1 shows, GEHIP determinants of morbidity are posited to arise from preventive health care effects while GEHIP curative care effects arise from improvements in the quality and accessibility of curative care. GEHIP is known to have had substantial mortality effects, concentrated among neonates [23]. What is less clear, however, is whether GEHIP achieved this result solely by improving care for sick children, or through morbidity effects associated with improvements in preventive health services (Fig 1 pathway labeled “***A***”). Results showing nil morbidity effects would suggest that the observed GEHIP mortality result is attributable to curative care impact only, while significant morbidity reduction effects would be indicative of an association of GEHIP exposure with preventive health care improvement. This paper analyses the impact of GEHIP on childhood morbidity using three of the most common under-five ill health conditions: diarrhea, fever, and cough.

## 3. Methodology

### 3.1. Data sources

GEHIP conducted two rounds of cross-sectional surveys of households in the UER treatment and comparison districts. The baseline survey antedated the start of the implementation of the GEHIP interventions in 2011 and the endline survey was conducted at the end of the project in late 2015. Both rounds shared an identical two-stage sampling procedure and questionnaire design. In the first stage of the baseline, 66 enumeration areas (EAs) or clusters were randomly sampled from the 11 districts of the Upper East using population proportional to size of the districts. To enhance statistical efficiency, clusters sampled for the baseline were reused in the endline. A complete listing of all households in the sampled EAs was conducted to serve as sampling frame for second stage sampling. In the second stage, random household selection proceeded within each cluster proportional to enumeration area size until the target sample total of 6000 women of reproductive age were selected. A similar procedure was adopted at the end line with a complete re-listing of households in the 66 baseline clusters before all sampling from listed households. All women in their reproductive age (15–49 years) were interviewed.

In both the baseline and endline surveys, women were asked about the health status of children born in the last five years. Specific questions were asked about specific common childhood illnesses such as cough, diarrhea and fever. The questions were explicit that for each woman, questions were being asked about their own biological children (i.e., children they have given birth to). We use three main indicators of childhood morbidity. They are maternal retrospective survey indicator variables for maternal recall of an own child having experienced i) cough, ii) diarrhea or iii) fever in the last two weeks. Our analysis is restricted to the sample of all children under five years born to the women at both baseline and endline.

### 3.2. Statistical methods

GEHIP aimed to test the hypothesis that health systems strengthening at the *district level* improves child health by reducing childhood morbidity. Strengthening the health systems, as articulated by GEHIP, is complex, multidimensional, and involves various health sector players–leadership and governance, workforce performance, information generation and utilization, health financing, essential drug supply and overall performance. The GEHIP project was designed to accommodate systems analyses that bring into account the multi-level aspects of the administrative hierarchy of the program, the research and the health and survival implications of household exposure to services at different levels of the health delivery system.

A regression specification of the Heckman (1974) difference-in- difference (DiD) procedure for calculating average treatment effects (ATE) [24] is given by:

$${morbidity}_{idt}=\alpha +\gamma {GEHIP}_{d}+\theta {end}_{t}+\beta ({GEHIP}_{id}*{end}_{idt})+X_{idt}^{\prime }\rho +\varepsilon_{idt}$$ (1)

where i denotes child, d denotes district and t denotes time (survey round). GEHIP is an indicator of whether a cluster is located in a GEHIP intervention district, *end* is an indicator for whether a given observation is from the endline survey, and X denotes a vector of potentially confounding maternal and household characteristics that predict child morbidity.

In this analysis, we include child age and gender, a dummy variable for whether the child was part of a multiple birth or not, maternal age, education, marital status, health insurance status, month of birth of child, a household-level five-category principal component score wealth index, occupation, religion and ethnicity. The parameter *ε* is the error term. The primary parameter of interest is β, an interaction term that estimates the DiD effect. It measures additional reduction in child morbidity in the GEHIP intervention districts over and above any reduction in the comparison districts that is attributed to GEHIP interventions. Because our sample includes multiple children from the same mother in a particular household, in all our estimations, we adjust our standard errors at the household level to account for potentially correlated error in the outcome variables among children of the same mother and belonging to the same household.

The study protocol was approved by the Ethical Review Committee of the Ghana Health Service, the Institutional Review Board (IRB) of the Navrongo Health Research Centre and the ethical review board of the Columbia University Medical Center, Mailman School of Public Health. Written Informed consent was obtained from all study participants, that is, women aged 15–49 years. Enumerators read the written informed consent form to participants in their local language and explained its content before participants decided whether to participate endorsed two copies of the form and a copy was given to the participant. The study followed all protocols as approved by the three ethical review boards.

## 4. Results

### 4.1. Descriptive statistics

Table 1 presents the descriptive statistics on the children included in the analysis. Both baseline and endline descriptive information are disaggregated by GEHIP intervention status. Our analysis sample includes 2697 children born to 2697 women from 2420 households at the baseline and 3922 children born to 2984 women from 2719 households at the endline. In the baseline 235 households (140 in treatment and 95 in comparison) had more than one eligible woman and each eligible woman contributed exactly one child under 5 years. At the endline, 958 households (476 in intervention and 482 in comparison) contributed more than one eligible woman while 869 women (433 in the intervention and 436 in the comparison) contributed more than one child under five years to the sample. In both periods, the numbers of children are almost evenly distributed between the intervention and comparison districts.

**Table 1:** Descriptive statistics.

Characteristic	Baseline		End line	
	Intervention	Comparison	Intervention	Comparison
Number of children	1345	1352	1980	1942
Number of women	1345	1352	1514	1470
Number of households	1179	1241	1403	1316
Sex of child				
Boy	52.18%	51.63%	52.32%	50.00%
Girl	47.27%	48.37%	47.69%	50.00%
Child is singleton				
Yes	3.94%	3.55%	11.41%	14.34%
No	96.06%	96.45%	88.59%	85.66%
Age of child in years (std dev)	1.84(1.37)	1.82(1.40)	1.93(1.42)	1.91(1.40)
Maternal age				
15–19 years	5.71%	5.79%	3.69%	4.58%
20–24 years	19.25%	23.08%	17.98%	22.50%
25–29 years	24.23%	22.28%	23.18%	25.28%
30–34 years	21.37%	22.13%	21.21%	20.39%
35–39 years	16.80%	16.02%	19.34%	15.19%
40–44 years	9.87%	8.33%	10.96%	9.01%
45–49 years	2.77%	2.38%	3.64%	3.04%
Maternal education				
No formal education	67.90%	74.71%	67.53%	70.55%
Primary/Middle school/JHS	28.06%	22.56%	25.96%	24.92%
Secondary plus	4.03%	2.73%	6.52%	4.53%
Maternal marital status				
Never married	3.57%	2.83%	2.78%	3.04%
Married	92.13%	93.54%	91.46%	92.38%
Divorced/widowed/separated	4.30%	3.62%	5.76%	4.58%
Maternal marital status				
Never married	3.57%	2.83%	2.78%	3.04%
Married	92.13%	93.54%	91.46%	92.38%
Divorced/widowed/separated	4.30%	3.62%	5.76%	4.58%
Maternal religion				
Christian	55.08%	45.86%	56.46%	53.24%
Traditional	17.26%	14.92%	11.01%	10.56%
Islam	24.19%	35.23%	31.11%	33.06%
No religion	3.47%	3.98%	1.41%	3.14%
Maternal ethnicity				
Buli	30.73%	0.16%	29.44%	0.15%
Frafra	26.05%	40.91%	21.36%	47.63%
Kusasi	19.11%	39.73%	20.40%	33.37%
Others	24.11%	19.20%	28.79%	18.85%
Maternal occupation				
No Occupation			10.71%	12.87%
Farming	52.66%	41.17%	49.65%	34.14%
Trading	20.32%	19.38%	16.52%	21.42%
Hairdressing/dressmaking	10.40%	15.47%	13.69%	14.78%
Student	1.53%	1.80%	1.72%	1.65%
Others	15.08%	22.19%	7.73%	15.14%
Wealth Index				
Poorest	23.27%	16.18%	19.29%	16.00%
Poor	19.63%	20.19%	22.68%	19.25%
Better	16.88%	21.83%	23.43%	19.35%
Less poor	20.74%	22.05%	19.90%	21.52%
Least poor	19.48%	19.75%	14.70%	23.89%
Had diarrhea in the last two weeks				
Yes	20.78%	16.40%	7.77%	10.15%
No	79.22%	83.60%	92.23%	89.85%
Had fever in the last two weeks				
Yes	10.79%	9.46%	6.89%	13.36%
No	89.11%	90.54%	93.11%	86.64%
Had cough in the last two weeks				
Yes	18.21%	13.05%	14.90%	17.73%
No	81.79%	86.95%	85.10%	82.27%

Table 1 shows that the respondents are generally youthful. At the baseline, about 49% of women in the intervention districts and about 51% of women in the comparison areas were under the age of 30 years. The overall age distribution was similar between intervention and comparison districts. At the endline, 45% of women in the intervention areas and 52% of women in the comparison areas were below 30 years. Overall, educational attainment of the women was very low even though it was slightly better in the comparison group. About 68% percent women in the intervention areas and 75% of women in the comparison areas had no formal education, with less than 4% of the overall sample having completed secondary education or higher. Educational attainment improved only slightly at the endline. An overwhelming majority of children in our sample were born to mothers who are currently married. This was similar between the intervention and comparison areas at both baseline and endline. More than half of the women in our sample were not covered under health insurance. Insurance coverage improved at endline, although the improvement in intervention areas was more than that of comparison areas. Majority of the women are Christians. In terms of occupation, farming is the most common occupation among the women whose children are included in our sample. Table 1 shows that at baseline, more women in the intervention areas belonged to poor wealth quintiles compared to women from comparison areas and this was similar at endline.

### 4.2. Difference-in-difference analyses

**Fig 2:**
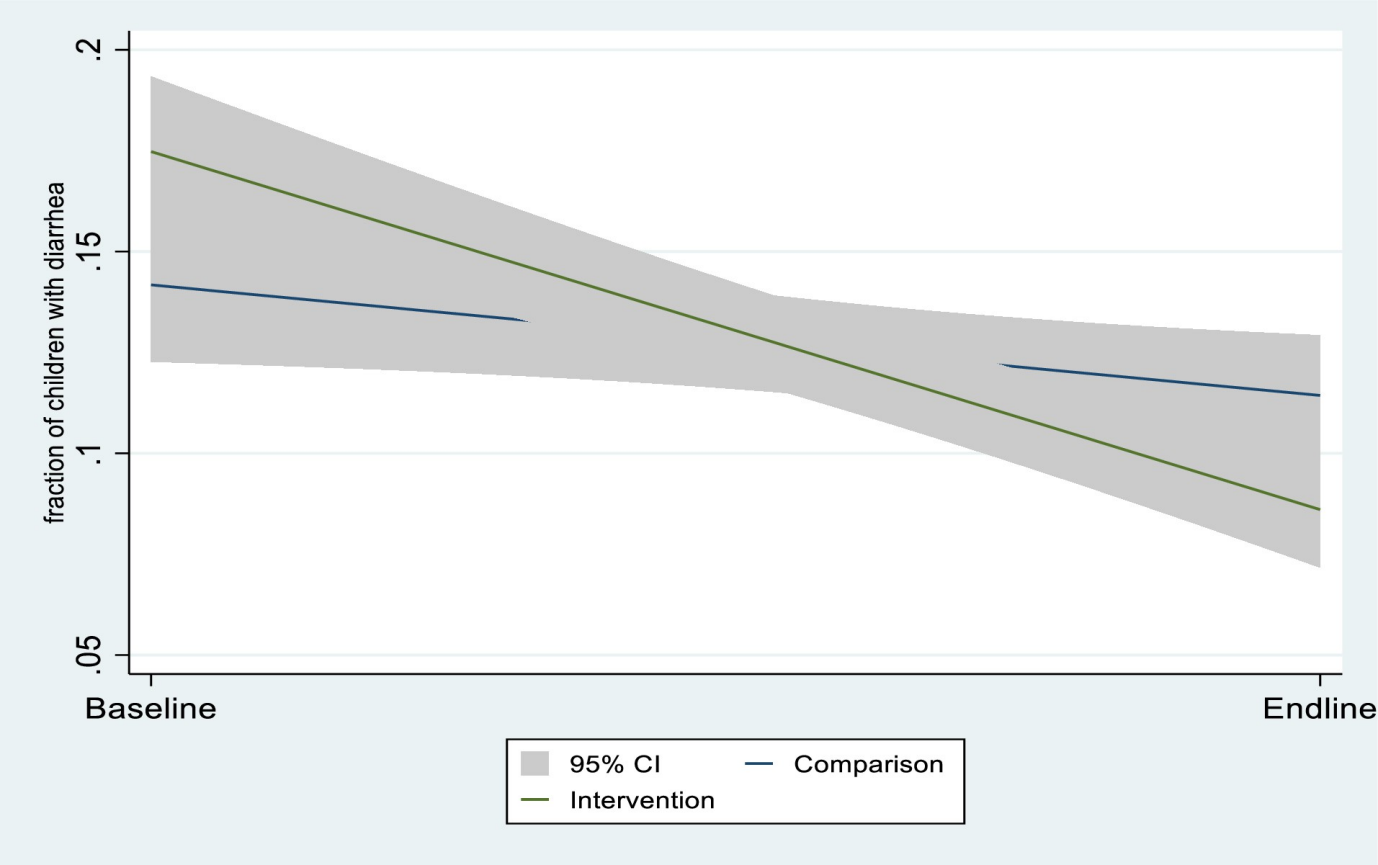
The effect of GEHIP on the incidence of childhood diarrhea, according to maternal recall.

**Fig 3:**
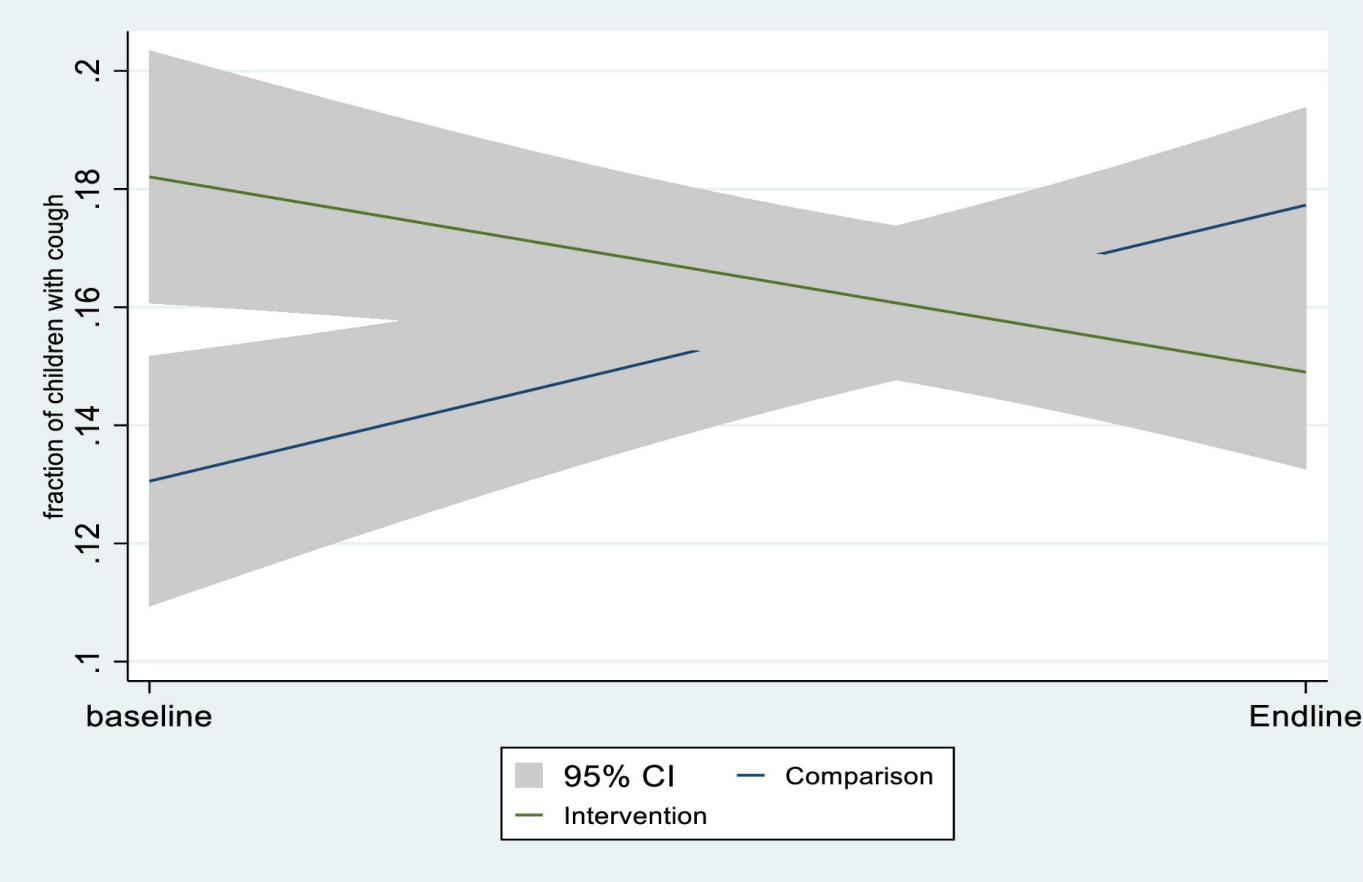
The effect of GEHIP on the incidence of childhood cough, according to maternal recall.

**Fig 4:**
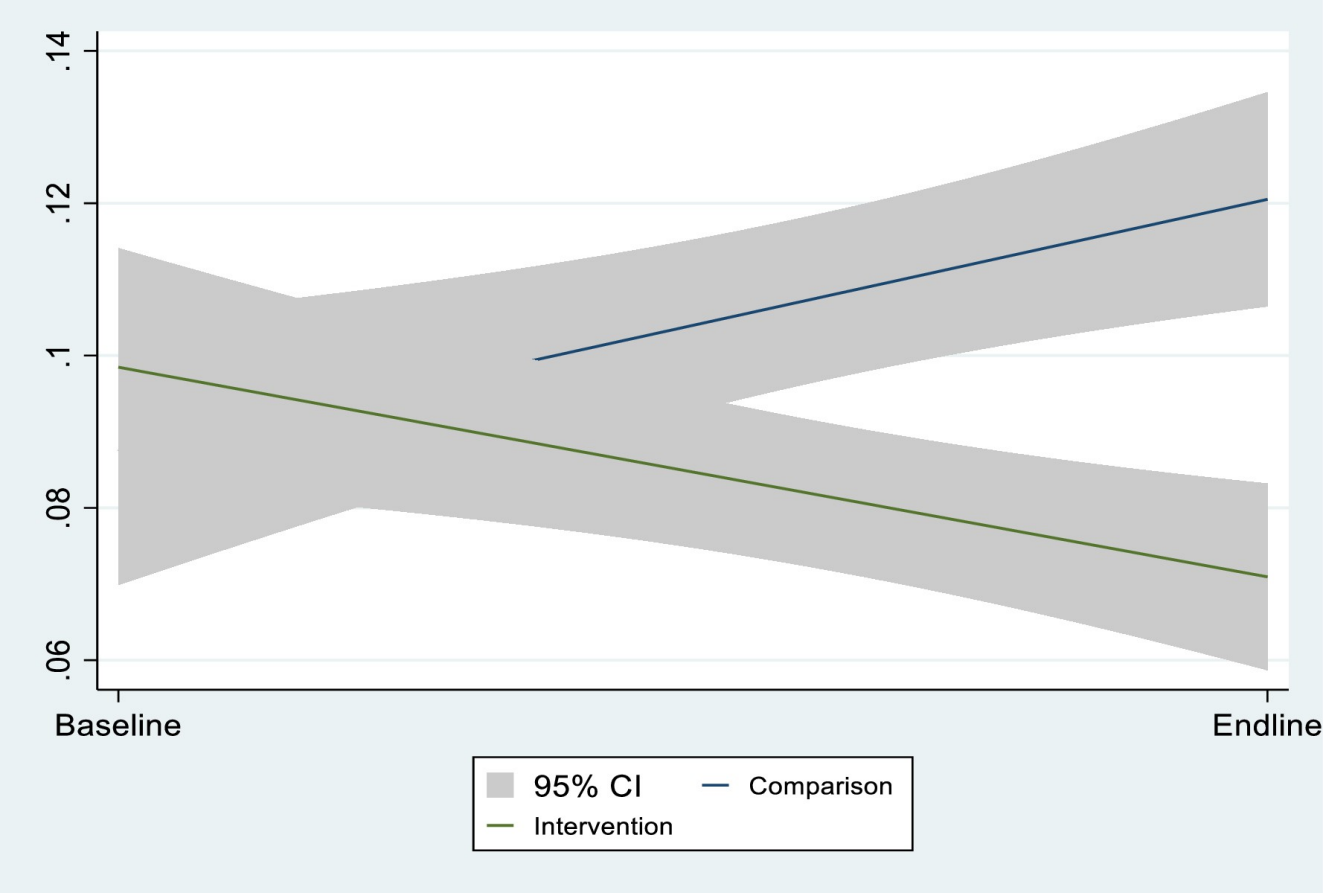
The effect of GEHIP on the incidence of childhood fever, according to maternal recall.

Figs 2–4 presents crude (unadjusted) graphical evidence of the effect of the GEHIP interventions on the three main outcome variables used in the analysis. Fig 2 shows the GEHIP difference-in-difference result for maternal recall of childhood diarrhea. Figs 3 and 4 show the corresponding effect for cough and fever.

Fig 2 shows that there is no evidence of an effect of GEHIP on the incidence of diarrhea but Figs 3 and 4 show that the GEHIP lead to statistically significant reductions in the likelihood that children experienced episodes of cough and fever in the preceding two weeks.

### 4.3. Regression results

Tables 2–4 present regression results for the model 1 estimation of the effect of GEHIP on childhood morbidity (diarrhea, fever and cough respectively). Each table reports odd-ratios (OR) and 95% confidence interval from a logistic regression on the effect of GEHIP on the incidence of childhood diarrhea, fever and cough respectively. It must be noted that the level of significance for interpreting the results of the analysis is defined as <0.05. For Table 2, Column 2 presents the results for diarrhea. The OR is 0.616 and although it is not statistically significant, it shows a positive impact of GEHIP on the incidence of childhood diarrhea (38% reduction). Girls and older children were less likely to have experienced diarrhea while respondents were more likely to report that a child had diarrhea if they are born to Frafra mothers compared with those born to Buli mothers.

**Table 2:** The estimated effect of GEHIP on health system childhood morbidity (Diarrhea).

VARIABLES	Diarrhea	
	Odds-ratio	95% CI
Intervention	1.564***	(1.123–2.177)
End line	0.545**	(0.312–0.952)
Intervention*End line	0.616*	(0.379–1.001)
Child is less than 1 year (ref)	1.000	
Child is 1–2 years	1.388***	(1.136–1.696)
Child is 2–3 years	1.000	(0.758–1.320)
Child is 3–4 years	0.868	(0.669–1.126)
Child is 4–5 years	0.781*	(0.586–1.042)
Child is a boy (ref)	1.000	
Child is a girl	0.845**	(0.715–0.999)
Child is a singleton (ref)	1.000	
Child was a multiple birth	1.062	(0.661–1.708)
Mother’s age: under 19 (ref)	1.000	
Mother’s age: 20–24	0.873	(0.579–1.316)
Mother’s age: 25–29	0.832	(0.534–1.295)
Mother’s age: 30–34	0.658	(0.396–1.093)
Mother’s age: 35–39	0.668	(0.401–1.111)
Mother’s age: 40–44	0.633*	(0.368–1.087)
Mother’s age: 45–49	0.498*	(0.232–1.068)
Mother is never married	1.000	
Mother currently married	1.012	(0.655–1.562)
Mother previously married	1.343	(0.766–2.354)
Mother’s education: none (ref)	1.000	
Mother education: Primary/JHS	1.021	(0.797–1.308)
Mother’s education: Secondary	0.734*	(0.524–1.027)
Mother not enrolled in health insurance (ref)	1.000	
Mother enrolled in health insurance	0.863	(0.683–1.089)
Wealth index: poorest (ref)	1.000	
Wealth index: poorer	0.919	(0.721–1.171)
Wealth index: middle	0.846	(0.630–1.138)
Wealth index: richer	0.963	(0.713–1.301)
Wealth index: richest	1.057	(0.751–1.486)
Mother’s occupation: None (ref)	1.000	
Mother’s occupation: farming	1.086	(0.784–1.507)
Mother’s occupation: Trading	1.338	(0.931–1.922)
Mother’s occupation: hairdress	1.299	(0.887–1.900)
Mother’s occupation: Student	0.975	(0.481–1.975)
Mother’s occupation: other	1.183	(0.820–1.707)
Mother’s religion: Christian (ref)	1.000	
Mother’s religion: Traditional	0.989	(0.728–1.344)
Mother’s religion: Muslim	1.201	(0.915–1.576)
Mother’s religion: No religion	0.769	(0.418–1.415)
Ethnicity: Buli	1.000	
Ethnicity: Frafra	1.884***	(1.364–2.603)
Ethnicity: Kusasi	1.350	(0.886–2.056)
Ethnicity: Others	1.349	(0.846–2.153)
Constant	0.123***	(0.054–0.281)
Observations	5,783	
Pseudo-R^2^	0.0358	
Wald Chi-square	393.97	

Notes: OR is Odd-ratios; 95% CI is 95% confidence interval. Table reports results from logistic regression model. The outcome variable is a dummy variable that takes of value of 1 if the child had diarrhea in the preceding two weeks or zero otherwise. The regression controls for month of birth dummies but the coefficients are not reported. Standard errors are clustered at the enumeration area level.

*** p<0.01

** p<0.05

* p<0.1.

**Table 3:** The estimated effect of GEHIP on health system childhood morbidity (Fever).

VARIABLES	Fever	
	Odds-ratio	95% CI
Intervention	1.278	(0.892–1.832)
End line	0.328***	(0.163–0.662)
Intervention*End line	0.552**	(0.310–0.982)
Child is less than 1 year (ref)	1.000	
Child is 1–2 years	1.440***	(1.108–1.872)
Child is 2–3 years	1.494***	(1.111–2.010)
Child is 3–4 years	1.472***	(1.100–1.969)
Child is 4–5 years	1.359**	(1.041–1.773)
Child is a boy (ref)	1.000	
Child is a girl	0.971	(0.818–1.154)
Child is a singleton (ref)	1.000	
Child was a multiple birth	1.419	(0.786–2.561)
Mother’s age: under 19 (ref)	1.000	
Mother’s age: 20–24	1.125	(0.652–1.940)
Mother’s age: 25–29	1.167	(0.690–1.975)
Mother’s age: 30–34	1.151	(0.701–1.890)
Mother’s age: 35–39	1.221	(0.726–2.051)
Mother’s age: 40–44	1.371	(0.742–2.533)
Mother’s age: 45–49	1.348	(0.693–2.622)
Mother is never married	1.000	
Mother currently married	0.933	(0.472–1.847)
Mother previously married	1.194	(0.540–2.640)
Mother’s education: none (ref)	1.000	
Mother education: Primary/JHS	1.190	(0.912–1.553)
Mother’s education: Secondary	1.222	(0.687–2.173)
Mother not enrolled in health insurance (ref)	1.000	
Mother enrolled in health insurance	1.047	(0.813–1.347)
Wealth index: poorest (ref)	1.000	
Wealth index: poorer	0.910	(0.697–1.189)
Wealth index: middle	0.845	(0.589–1.214)
Wealth index: richer	0.874	(0.637–1.199)
Wealth index: richest	0.930	(0.650–1.329)
Mother’s occupation: None (ref)	1.000	
Mother’s occupation: farming	0.919	(0.645–1.309)
Mother’s occupation: Trading	0.937	(0.604–1.456)
Mother’s occupation: hairdress	1.019	(0.662–1.567)
Mother’s occupation: Student	0.746	(0.261–2.127)
Mother’s occupation: other	0.848	(0.546–1.319)
Mother’s religion: Christian (ref)	1.000	
Mother’s religion: Traditional	0.764	(0.514–1.134)
Mother’s religion: Muslim	1.317**	(1.023–1.696)
Mother’s religion: No religion	0.796	(0.385–1.645)
Ethnicity: Buli	1.000	
Ethnicity: Frafra	1.442	(0.926–2.246)
Ethnicity: Kusasi	1.241	(0.771–1.998)
Ethnicity: Others	1.378	(0.838–2.265)
Constant	0.077***	(0.027–0.220)
Observations	5,806	
Pseudo-R^2^	0.0374	
Wald Chi-Square	504.60	

Notes: OR is Odd-ratios; 95% CI is 95% confidence interval. Table reports results from logistic regression model. The outcome variable is a dummy variable that takes of value of 1 if the child had malaria in the preceding two weeks or zero otherwise. The regression controls for month of birth dummies but the coefficients are not reported. Standard errors are clustered at the enumeration area level.

*** p<0.01

** p<0.05

* p<0.1.

**Table 4:** The estimated effect of GEHIP on health system childhood morbidity (Cough).

VARIABLES	Cough	
	Odds-ratio	95% CI
Intervention	1.949***	(1.396–2.721)
End line	0.284***	(0.155–0.520)
Intervention*End line	0.531**	(0.299–0.942)
Child is less than 1 year (ref)		
Child is 1–2 years	1.404***	(1.122–1.755)
Child is 2–3 years	1.157	(0.937–1.428)
Child is 3–4 years	1.044	(0.853–1.277)
Child is 4–5 years	1.131	(0.894–1.431)
Child is a boy (ref)		
Child is a girl	1.043	(0.922–1.181)
Child is a singleton (ref)		
Child was a multiple birth	0.920	(0.610–1.385)
Mother’s age: under 19 (ref)		
Mother’s age: 20–24	1.141	(0.770–1.691)
Mother’s age: 25–29	1.033	(0.684–1.561)
Mother’s age: 30–34	0.863	(0.554–1.344)
Mother’s age: 35–39	0.992	(0.629–1.566)
Mother’s age: 40–44	0.974	(0.611–1.552)
Mother’s age: 45–49	0.888	(0.440–1.795)
Mother is never married		
Mother currently married	1.380	(0.731–2.604)
Mother previously married	1.121	(0.549–2.290)
Mother’s education: none (ref)		
Mother education: Primary/JHS	1.272**	(1.052–1.538)
Mother’s education: Secondary	0.977	(0.638–1.496)
Mother not enrolled in health insurance (ref)		
Mother enrolled in health insurance	1.201**	(1.015–1.421)
Wealth index: poorest (ref)		
Wealth index: poorer	1.166	(0.858–1.584)
Wealth index: middle	1.194	(0.879–1.623)
Wealth index: richer	1.217	(0.869–1.705)
Wealth index: richest	1.112	(0.737–1.679)
Mother’s occupation: None (ref)		
Mother’s occupation: farming	0.986	(0.660–1.473)
Mother’s occupation: Trading	1.097	(0.746–1.614)
Mother’s occupation: hairdress	1.353	(0.900–2.034)
Mother’s occupation: Student	0.569	(0.238–1.358)
Mother’s occupation: other	1.114	(0.721–1.720)
Mother’s religion: Christian (ref)		
Mother’s religion: Traditional	1.084	(0.832–1.412)
Mother’s religion: Muslim	1.283**	(1.029–1.599)
Mother’s religion: No religion	0.785	(0.388–1.588)
Ethnicity: Buli		
Ethnicity: Frafra	1.300*	(0.968–1.745)
Ethnicity: Kusasi	1.023	(0.677–1.544)
Ethnicity: Others	0.979	(0.637–1.505)
Constant	0.138***	(0.058–0.325)
Observations	5,758	
Pseudo-R^2^	0.0357	
Wald Chi-square	739.55	

Notes: OR is Odd-ratios; 95% CI is 95% confidence interval. Table reports results from logistic regression model. The outcome variable is a dummy variable that takes of value of 1 if the child had cough in the preceding two weeks or zero otherwise. The regression controls for month of birth dummies but the coefficients are not reported. Standard errors are clustered at the enumeration area level.

*** p<0.01

** p<0.05

* p<0.1.

For Table 3, Column 2 presents the results of the effect of GEHIP on fever. The ORs of the main parameter of interest is 0.552 and this is statistically significant at 5%. This means that GEHIP led to a statistically significant 45% reduction in the likelihood that children had fever in the preceding two weeks. Compared to children who are less than one year, the likelihood that children had fever in the preceding two weeks peaks at ages 2–3 and starts falling. Another factor that affects the likelihood of having fever is religion: Children born to Muslim mothers were more likely to have fever in the preceding two weeks compared to children born to Christian mothers.

For Table 4, Column 2 presents the results on the effect of GEHIP on cough. The OR of the parameter of interest is 0.531 and is statistically significant at the 5% confidence level. This means that GEHIP exposure was associated with a statistically significant 47% reduction in the likelihood of respondent reporting that a child had a cough in the preceding two weeks. The other significant predictors of the cough are child’s age, maternal education, health insurance status of mother, and religion. Younger children have a higher likelihood to have experienced cough while children born to mothers with basic education (primary and junior high school) were more likely to have experienced cough compared with those whose mothers had no education. Compared to mothers who were not enrolled in health insurance, children born to mothers enrolled in health insurance were more likely to have experienced cough. Again, children born to Muslim mothers were more likely to have cough in the last two weeks.

## 5. Discussion

This analysis has estimated the child morbidity effect of the GEHIP program. Previous publications have assessed the child mortality effect of GEHIP [23], and the implementation cost per capita of implementing GEHIP (26). These previous publications show that GEHIP achieved about 48% reduction in neonatal mortality and its implementation cost as a percentage of total primary healthcare cost was relatively low [23, 25]. However, the effect of GEHIP on child morbidity reduction has not been explored. As part of the evaluation of healthcare interventions, it is often as equally important to understand the intermediate outputs as much as the final outcomes. Moreover, establishing the association of GEHIP exposure with morbidity effects is indicative of an important preventive health care effect of the set of system strengthening interventions that GEHIP pursued. Results suggest that mortality effects were not solely an outcome of improved curative care; preventive care improvements that reduced morbidity contributed to GEHIP’s impact. Regression results show that for two out of the three morbidity conditions considered in this study, GEHIP had a statistically significant reduction over and above those observed in comparison districts (GEHIP reduced the incidence of fever by 45% and cough by 47%).

In general, the results suggest that GEHIP’s sets of intervention had an equity effect because the reduction in morbidity for all three disease conditions were not statistically different across the five wealth indexes (socio-economic status). There was also no difference in the reduction of any of the diseases by occupational status. Research over the years has shown that addressing socio-economic differentials persists as the most challenging barrier to maternal and child mortality reduction in developing countries [26, 27]. The absence of differentials in the reduction by household socioeconomic status and occupational status of mothers is indicative of GEHIP morbidity reduction equity effects.

Our results are consistent with previous studies that have documented morbidity effects of health systems interventions in low-income countries [28]. Although studies documenting the effect health system interventions targeting all six pillars of the WHO are rare, there are several studies that have demonstrated strong effects of an integrated package of interventions on health outcomes [29–31]. Others have demonstrated strong morbidity impacts of various individual interventions [32, 33]. While identifying the contribution of the individual components of the package of interventions under GEHIP program would have been interesting and of relevance to policy makers, it is important to note that the nature of the rollout of the intervention under GEHIP does not permit us to do this. This is one important limitation of this paper.

## 6. Conclusion and policy implications

Developing community-based primary health care and strengthening health systems along the six pillars developed by the WHO are now globally recognized as a key strategy for achieving universal health coverage and attaining the SDGs. The GEHIP program in rural northern Ghana allowed us to test the proposition that a health system strengthening program could improve childhood health. Previous analysis of the GEHIP program show that the interventions led to significant reduction in child mortality. The results presented here show that in addition to these benefits, the GEHIP interventions led to large and significant reductions in the childhood morbidity and apparently had equity benefits, as well. GEHIP results show that an effort to strengthen a health system with interventions that expand the coverage of community-based primary health care and improve the range and quality of the services provided can reduce childhood morbidity.

## Supporting information

S1 File (DTA)

S2 File (DO)
